# Middle-Aged Men With HIV Have Diminished Accelerometry-Based Activity Profiles Despite Similar Lab-Measured Gait Speed: Pilot Study

**DOI:** 10.2196/11190

**Published:** 2019-02-01

**Authors:** Timothy M Hale, Viola Guardigni, Eva Roitmann, Matthieu Vegreville, Brooke Brawley, Erin Woodbury, Thomas W Storer, Paul E Sax, Monty Montano

**Affiliations:** 1 Partners HealthCare Pivot Labs Boston, MA United States; 2 Harvard Medical School Boston, MA United States; 3 S.Orsola-Malpighi Hospital Department of Medical and Surgical Sciences-DIMEC University of Bologna Bologna Italy; 4 Brigham & Women's Hospital Boston, MA United States; 5 Withings Paris France

**Keywords:** aging, digital biomarker, gait speed, HIV

## Abstract

**Background:**

People aging with HIV are living with increased risk for functional decline compared with uninfected adults of the same age. Early preclinical changes in biomarkers in middle-aged individuals at risk for mobility and functional decline are needed.

**Objective:**

This pilot study aims to compare measures of free-living activity with lab-based measures. In addition, we aim to examine differences in the activity level and patterns by HIV status.

**Methods:**

Forty-six men (23 HIV+, 23 HIV−) currently in the MATCH (Muscle and Aging Treated Chronic HIV) cohort study wore a consumer-grade wristband accelerometer continuously for 3 weeks. We used free-living activity to calculate the gait speed and time spent at different activity intensities. Accelerometer data were compared with lab-based gait speed using the 6-minute walk test (6-MWT). Plasma biomarkers were measured and biobehavioral questionnaires were administered.

**Results:**

HIV+ men more often lived alone (*P*=.02), reported more pain (*P*=.02), and fatigue (*P*=.048). In addition, HIV+ men had lower blood CD4/CD8 ratios (*P*<.001) and higher Veterans Aging Cohort Study Index scores (*P*=.04) and T-cell activation (*P*<.001) but did not differ in levels of inflammation (*P*=.30) or testosterone (*P*=.83). For all participants, accelerometer-based gait speed was significantly lower than the lab-based 6-MWT gait speed (*P*<.001). Moreover, accelerometer-based gait speed was significantly lower in HIV+ participants (*P*=.04) despite the absence of differences in the lab-based 6-MWT (*P*=.39). HIV+ participants spent more time in the lowest quartile of activity compared with uninfected (*P*=.01), who spent more time in the middle quartiles of activity (*P*=.02).

**Conclusions:**

Accelerometer-based assessment of gait speed and activity patterns are lower for asymptomatic men living with HIV compared with uninfected controls and may be useful as preclinical digital biomarkers that precede differences captured in lab-based measures.

## Introduction

Survival for people aging with HIV (PAWH) is now approaching the lifespan of uninfected individuals, in part, owing to the success of effective antiretroviral therapy [1]. In developed countries, more than half of the PAWH are over 50 years old and may be at greater risk for age-related comorbidities, including a decline in functional capacity [2]. Why these age-related comorbidities more often occur at younger ages in PAWH compared with uninfected (HIV−) individuals is presently unclear [3].

The functional decline in PAWH increases their risk for premature frailty and early mortality [4,5]. In multiple studies, slower walking speed has been consistently associated with a decline in mobility and performance of activities of daily living [6-8], and faster decline in walking speed is associated with increased mortality [9]. An emerging challenge is that while current assessments of functional capacity and mobility limitations in PAWH aged <65 years have been effective in identifying impairment [5,10], these assessment tools were historically developed in the context of geriatric population studies of adults aged >65 years [7] and may not always be suitable for assessing middle-aged individuals without overt mobility limitations [11]. Therefore, there is an unmet need for additional diagnostic tools sensitive to preclinical risk factors for the functional decline that occurs in middle-aged (ie, 50-65 years) persons [12]. One such tool might be physical activity monitoring. People living with HIV tend to be sedentary and less fit [13,14]. In this study, we posited that the objective assessment of habitual physical activity might provide a more sensitive way to identify preclinical changes in middle-aged HIV+ men.

Wearable accelerometers are now frequently used in research studies to measure free-living physical activity and are useful in providing an objective measurement of low-intensity activity [15,16]. The use of accelerometry in the function assessment of PAWH is limited [17-21]. The use of consumer-grade accelerometers has become cost-effective [22]. Although technical and methodological limits remain [16,22], many consumer-grade accelerometers display strong validity when compared with lab-based measurements and research-grade accelerometers [23,24].

This study included participants in the MATCH (Muscle and Aging in Treated Chronic HIV) cohort of middle-aged men and women living with HIV and uninfected control participants of similar age [25]. Overall, HIV+ participants in the MATCH cohort are relatively healthy but displayed increased levels of inflammation and immune activation compared with their uninfected counterparts and displayed modest but significant subclinical deficits in lab-based physical function [25]. However, when men and women were analyzed separately, we noticed that performance in the lab-based 6-minute walk test (6-MWT) in men did not reach significance despite reporting greater fatigue [25]. We hypothesized that measuring physical activity in daily life using consumer-grade accelerometers might provide additional information, potentially detecting subclinical deficits that do not as yet reach significance in the lab-based assessment of gait speed. Therefore, this pilot study aims to measure and compare walking speed and activity patterns between HIV+ and HIV− men in the MATCH cohort using a consumer-grade accelerometer. Accelerometer data were used to measure steps taken per minute and to estimate the gait speed and classify patterns of activity.

## Methods

### Study Population

Participants in this study (MPACT: MATCH Physical ACtivity Tracker) consisted of HIV+ and HIV− men aged 50-65 years who were enrolled in the MATCH study [25] and who consented to this study. The MATCH cohort is a longitudinal observational study of middle-aged people living with HIV on effective ART, along with age-matched uninfected controls, all living in the Boston metropolitan area in Massachusetts [25]. At the first visit, the study protocol and procedures were explained and consent obtained. After consent, demographic information was collected, and questionnaires were administered to assess health-related quality of life (HRQoL; Patient Reported Outcome Measurements Information System [PROMIS-29]), disability (Pepper Assessment Tool for Disability [PAT-D]), and depression (Center for Epidemiological Studies Depression [CES-D]). All study procedures were approved by the Partners Human Research Committee institutional review board.

### Questionnaires

#### Patient Reported Outcome Measurements Information System-29

PROMIS-29 assesses general HRQoL on 7 dimensions, including depression, anxiety, physical function, pain, fatigue, sleep, and social engagement with each of the 7 domains having 4 questions and a single question assessing pain intensity [26]. For example, in the pain domain, a sample question is, “*How much did pain interfere with your day to day activities?*” Responses include a range of 5 choices (eg, not at all, very much). The pain intensity is assessed by, *In the past 7 days, how would you rate your pain on average?* Responses range from 0 “No pain” to 10 “Worst imaginable pain.” Scores are negatively associated with HRQoL.

#### Pepper Assessment Tool for Disability

The PAT-D is a 19-item questionnaire used to assess whether functional limitations impact disability across 3 domains—mobility, activities of daily living, and instrumental activities of daily living [27]. Responses are recorded on a 5-point Likert scale. An example question is, “*How much difficulty walking one block?*” Responses include a range of 7 choices (eg, no difficulty, a lot of difficulty).

#### Center for Epidemiological Studies Depression

The CES-D scale measures clinical depression [28] and uses a 20-item score to assess clinically significant depression [29]. An example question is, “*How often over the past week were you depressed?*” Responses include a range of 4 choices (eg, rarely, most or all the time).

#### Functional Assessment of Chronic Illness Therapy-Fatigue

Fatigue was assessed using the 13-item Functional Assessment of Chronic Illness Therapy-Fatigue (FACIT-F) subscale [30]. An example question is, “*How often do you feel fatigued?*” Responses include a range of 5 choices (eg, not at all, very much).

### Veterans Aging Cohort Study Index Score and Biomarkers

The Veterans Aging Cohort Study Index calculation includes age, CD4 (cluster of differentiation 4 T cells), HIV RNA, Hb, creatinine, FIB-4, and HCV [31,32]. Blood biomarkers (CD4 T-cell count, CD4/CD8 T-cell ratio), an inflammatory composite score of circulating biomarkers for inflammation (ie, sCD163, sCD14, C-reactive protein, and interleukin-6 expression) and a T-cell immune activation composite score (ie, CD8, CD38, and HLA-DR expression) were evaluated in this study sample similar to what is described for the larger cohort [25]. Total testosterone (TT) and serum hormone-binding globulin (SHBG) were measured in the Brigham Research Assay Core facility using liquid chromatographic-tandem mass spectrometry (LC-MS/MS). SHBG was measured using radioimmunoassay. Free testosterone (FT) was calculated from TT and SHBG [33].

### Accelerometer Data Collection and Measures

The accelerometer used in this study was the Withings Pulse Ox, a consumer-grade triaxial accelerometer with published validity for step counts when compared with research-grade accelerometers [23,24]. Participants were instructed to wear the accelerometer on their nondominant wrist 24 hours per day, 7 days per week for 3 consecutive weeks. Each individual in this study returned to the study center weekly for study personnel to recharge the Pulse Ox battery and upload data using a randomly assigned and anonymous study account. Data were collected in 1-minute intervals when any step activity was detected by the accelerometer, including low-intensity levels of activity that are not typically analyzed or accessible using consumer-grade trackers. In 13 cases, the tracker was lost or malfunctioned during the study period and was replaced to continue with one additional week of data collection. In 10 cases, data were collected for <3 weeks, primarily because of participants failure to wear the device. In total, 3 weeks of data were collected from 36 participants, 2 weeks from 6 participants, and 1 week of data from 4 participants.

In addition to step counts, accelerometer data were used to calculate gait speed, metabolic equivalents (METs), and relative activity intensity based on each participant’s activity distribution throughout the day. The mean gait speed in meters/second was based on the number of steps per minute and a formula using participant’s height in meters: (steps×height×0.414)/60) [34]. METs were calculated using a formula developed by Withings: 1+1/2×speed (km/h)+0.086×speed (km/h)×slope (in %) (Eva Roitmann, Withings, email communication; March 10, 2017 and March 28, 2018).

The standardized 6-MWT [11] was administered at the study site by the same trained and experienced exercise physiologist as in this study. A comparison test was conducted between both measures, in which participants wore an activity tracker (Pulse Ox) to determine the gait speed using the formula indicated above. The simultaneous measurement of gait speed using the lab-based assessment 6-MWT [calculated by the distance walked (m) in 6 minutes] and accelerometry indicated that the gait speed differed on average by <2%, similar to results in prior studies ([23,24] and Multimedia Appendix 1).

Accelerometer-derived patterns of physical activity were defined as the percent of the time a participant spends at one of 3 levels (ie, low, moderate, or intense) of their maximum observed intensity or gait speed. Two measures were created, a weekly measure using the relative maximum intensity observed for each participant during each week, and a summary measure that used the maximum observed intensity for a participant across 3 weeks of activity. The total activity for each individual was divided into 3 groups, with low activity defined as the lowest quartile (ie, 0%-25%, Q1), moderate activity as the middle 2 quartiles (ie, 25%-75%, Q2+Q3), and vigorous (intense) activity as the highest quartile (ie, 75%, Q4) for each participant.

### Statistical Analysis

Continuous and categorical variables were expressed as means (SD) or percentages. Differences in demographics, plasma biomarkers, and self-reported measures between HIV+ and HIV− men were analyzed by logistic regression and Student’s *t* tests. Differences by HIV status in accelerometer-derived measures of gait speed, METs, and activity patterns were hypothesized to be lower in HIV+ men and, therefore, conducted using a one-sided *t* test. One-sided *t* tests were used to analyze differences between groups (HIV+ vs HIV−) in lab-based physical function assessments of gait speed (6-MWT) and METs collected within the 16 months prior to this substudy [25]. Whether the HIV status and other markers were predictive of physical activity (ie, as gait speed assessment in the lab, free-living gait speed based on activity trackers, and lowest quartile of activity based on activity trackers) was assessed using univariate and multivariate linear regression. To evaluate what factors might be significant predictors of gait speed, the following variables were evaluated in univariate analysis: body mass index (BMI), HIV status, testosterone levels, and CD4/CD8 ratios. Multivariate models were used to examine BMI and HIV status as independent predictors of activity, and the significance of these predictors after adjusting for race, smoking, and living alone. An alpha of <.05 was used to indicate statistical significance.

## Results

### Characteristics of Study Participants

Forty-six men participating in the MATCH cohort [25] consented to participate in this substudy. Tables 1 and 2 present the characteristics of the study participants. Participants were aged between 50 and 65 years and included 23 asymptomatic, generally healthy HIV+ participants on effective antiretroviral therapy with undetectable viremia (<50 copies/mL) and 23 HIV− (confirmed by HIV serology) participants, all living in the Boston area. HIV+ participants were more often nonwhite (*P*=.001) and more often lived alone (*P*=.02). We observed no significant differences in education or working status. BMI and smoking behavior approach statistical significance with HIV+ men having a lower BMI compared with HIV− men (*P*=.05) and elevated smoking behavior (*P*=.51). Blood biomarker profiles indicated that when compared with HIV−, HIV+ participants had significantly reduced CD4/CD8 ratios (*P*<.001), higher Veterans Aging Cohort Study scores (*P*=.04), and elevated immune activation (*P*<.001) but did not differ in levels of inflammation (*P*=.30), TT (*P*=.83), FT (*P*=.72), or SHBG (*P*=.29). Biobehavioral questionnaires were administered to obtain self-reported measures and indicated that when compared with uninfected HIV− participants, HIV+ participants reported more fatigue (FACIT-F: *P*=.048) and pain intensity (PROMIS-29: *P*=.02); however, there were no self-reported differences in alcohol abuse (*P*=.79), depression (CES-D: *P*=.74), or disability (PAT-D: *P*=.39).

**Table 1:** The baseline characteristics of the study participants (continuous variables).

Continuous variables	HIV+ (n=23), mean (SD)	HIV− (n=23), mean (SD)	*P* value^a^
Age (years)	58.1 (3.8)	59.2 (4.5)	.38
Body mass index (kg/m^2^)	26.9 (3.8)	29.9 (5.8)	.05
CD4/CD8 ratio	0.99 (0.65)	2.80 (1.56)	<.001
Veterans Aging Cohort Study Index score	22.39 (8.48)	17.70 (6.49)	.04
Inflammation composite score^b^	1.09 (1.00)	0.78 (0.95)	.30
T-cell immune activation composite score^c^	1.43 (1.12)	0.09 (0.29)	<.001
Total testosterone^d^	2.82 (0.53)	2.78 (0.48)	.83
Free testosterone^d^	2.58 (0.56)	2.63 (0.43)	.72
Serum hormone-binding globulin^d^	4.00 (0.51)	3.84 (0.53)	.29
Fatigue (Functional Assessment of Chronic Illness Therapy-Fatigue)	41.91 (9.69)	46.61 (5.17)	.048
Pain intensity (Patient Reported Outcome Measurements Information System)^e^	3.22 (2.70)	1.52 (1.75)	.02
Disability (Pepper Assessment Tool for Disability)	1.38 (0.56)	1.26 (0.42)	.39

^a^*P* values were determined by 2-sided *t* tests.

^b^Composite score of sCD163, sCD14, CRP, and interleukin-6 expression.

^c^Composite score of CD8, CD38, and HLA-DR expression.

^d^Values are natural log-transformed.

^e^Scale 0 to 10, in the past 7 days.

**Table 2:** The baseline characteristics of the study participants (categorical variables).

Categorical variables	HIV+ (n=23), n (%)	HIV− (n=23), n (%)	*P* value^a^	Odds ratio (95% CI)
White	8 (35)	22 (96)	.001	0.02 (.00-.21)
Living alone	17 (77)^b^	10 (43)	.02	4.42 (1.21-16.12)
College degree	9 (41)^b^	13 (57)	.30	0.53 (.16-1.74)
Working	9 (39)	10 (43)	.77	0.84 (.26-2.71)
≥100 cigarettes lifetime	16 (73)^b^	10 (43)	.05	3.47 (.99-.12.09)
Alcohol abuse^c^	5 (23)^b^	6 (26)	.79	0.83 (.21-3.26)
Self-reported depression (Center for Epidemiological Studies Depression)^d^	7 (30)	6 (26)	.74	1.24 (.34-4.49)

^a^*P* values were determined by logistic regression with HIV status as the outcome.

^b^n=22.

^c^Defined as ≥3 drinks per day.

^d^CES-D score ≥16.

### Physical Function Assessment

Laboratory-based physical function assessment was recently described in MATCH participants [25], and those data were used to evaluate the subset of individuals in this study. In the subset of individuals in this study, no significant differences were detected between HIV+ and HIV− men in gait speed using the 6-MWT (*P*=.39), nor in predicted METs (*P*=.15; Table 3) [13]. However, all participants, both HIV+ and HIV−, displayed a significantly reduced accelerometer-based (aka, free-living or volitional) gait speed when compared with the lab-based 6-MWT measurements (0.44 [SD 0.13] m/s vs 1.52 [SD 0.27] m/s; *P*<.001). Furthermore, HIV+ participants displayed significantly slower free-living gait speeds (0.41 [SD 0.11] m/s vs 0.48 [SD 0.14] m/s; *P*=.04) and METs (1.03 [SD 0.31] vs 1.24 [SD 0.42]; *P*=.03) compared with HIV− participants under free-living conditions. The MET difference is equivalent to about 300 Kcal/day (based on 16 hours of waking time).

### Personalized Physical Activity Distribution

**Figure 1:**
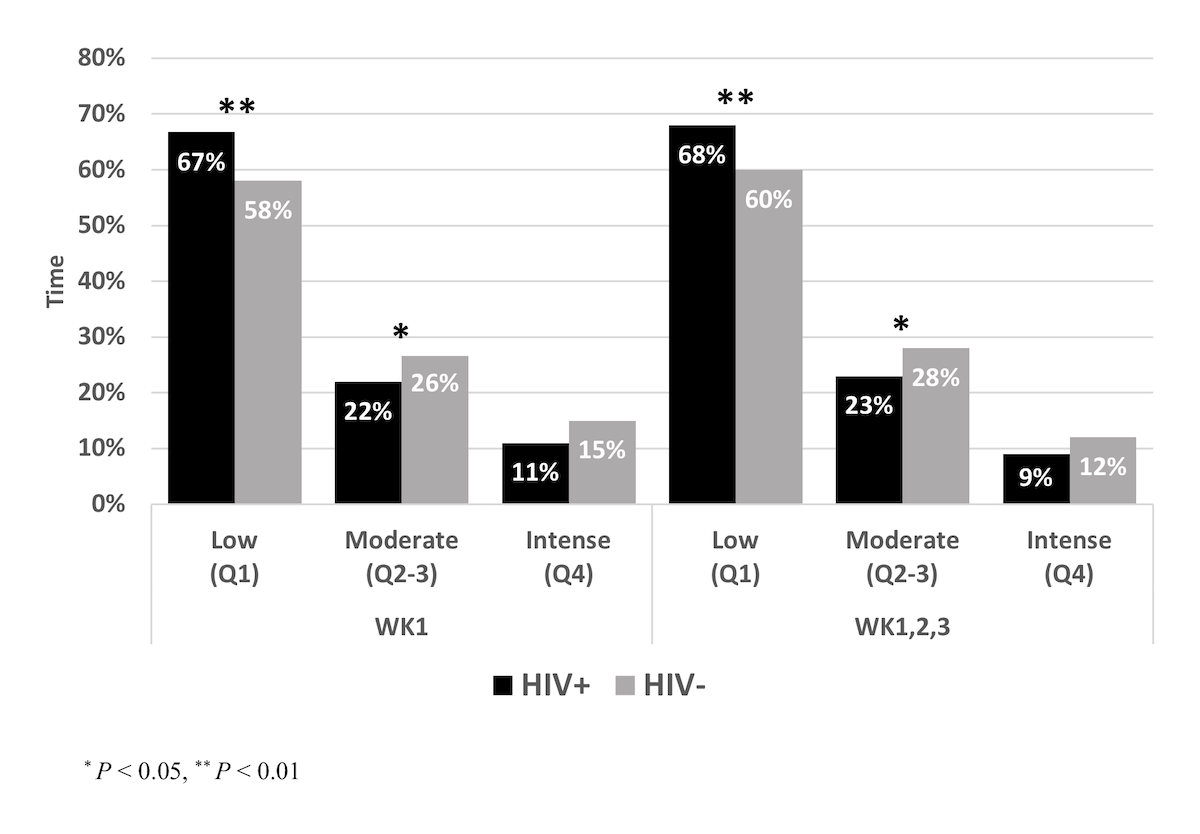
Comparison of free-living activity patterns between men living with HIV (HIV+) and uninfected men (HIV–), represented as mean percent time spent at different activity levels.

To evaluate patterns of daily activity, HIV+ participants were compared with HIV− participants based on the time spent as a percentage of the total activity for each participant. As shown in Figure 1, HIV+ participants spent significantly more of their time in the lowest quartile of activity compared with HIV− participants, during both the first week (*P*=.01) and all 3 weeks combined (*P*=.01; Table 3; Figure 1). By contrast, HIV− participants spent more of their time in the middle quartiles of activity compared with HIV+ participants, in the first week (*P*=.04) and again in all 3 weeks combined (*P*=.02; Table 3; Figure 1).

Tables 4 and 5 present the results from linear regression models. In univariate linear regression analyses, the BMI was the only significant predictor of lab-based gait speed (*P*=.04), whereas HIV status (*P*=.02), total and FT (*P*=.04), and the CD4/CD8 ratio (*P*=.02) were significant predictors of accelerometer-based metrics. In a multivariate regression model, the BMI and HIV status were independent predictors of accelerometer but not lab-based gait speed (Model 1). However, when adjusted for race, smoking, and living alone in Model 2, only BMI was an independent predictor of the accelerometer and lab-based gait speed, likely because of the small sample size of the study.

**Table 3:** Lab-based and accelerometer-based measurements of physical function and activity patterns.

Measurements			HIV+ (n=23)	HIV− (n=23)	*P* value^a^
**Lab-based assessments, mean (SD)**					
	6-minute walk test gait speed (m/s)		1.51 (0.23)	1.53 (0.31)	.39
	Predicted metabolic equivalents^b^		2.93 (0.30)	3.04 (0.41)	.15
**Accelerometer assessments, mean (SD)**					
	Gait speed (m/s)^c^		0.41 (0.11)	0.48 (0.14)	.04
	Predicted metabolic equivalents^c^		1.03 (0.31)	1.24 (0.42)	.03
	**Activity patterns^d^, n (%)**				
		Week 1: Q1 (Low, %)	67 (12)	58 (11)	.01
		Week 1: Q2-Q3 (Moderate, %)	22 (7)	26 (10)	.04
		Week 1: Q4 (Intense, %)	11 (9)	15 (11)	.11
		Week 1,2,3: Q1 (Low, %)	68 (13)	60 (10)	.01
		Week 1,2,3: Q2-Q3 (Moderate, %)	23 (7)	28 (10)	.02
		Week 1,2,3: Q4 (Intense, %)	9 (9)	12 (12)	.19

^a^*P* values were determined by one-sided *t* tests.

^a^Adjusted for height.

^c^Based on the following formula calculation for gait speed: ([steps×height×0.414]/60) [34]] and METs: 1+1/2×speed (km/h)+0.086×speed (km/h) ×slope (in %).

^d^Percent time spent in lowest (Q1), moderate (Q2-Q3), intense (Q4) quartiles of activity, as assessed using the maximum intensity from each week or from the overall period (21 days).

**Table 4:** Univariate linear regression models for activity outcomes.

Predictors^a^	Lab-based outcomes		Accelerometer-based outcomes			
	Gait speed		Gait speed		Low activity	
	Coefficient (95% CI)	*P* value	Coefficient (95% CI)	*P* value	Coefficient (95% CI)	*P* value
HIV status	−0.01 (−0.11 to 0.10)	.91	−0.15 (−0.34 to 0.03)	.10	0.16 (0.02 to 0.29)	.02
Body mass index	−0.32 (−0.63 to −0.02)	.04	−0.44 (−0.98 to 0.097)	.11	−0.06 (−0.50 to 0.38)	.78
Total testosterone	0.06 (−0.05 to 0.18)	.26	0.21 (0.02 to 0.40)	.04	−0.02 (−0.19 to 0.14)	.77
Free testosterone	0.05 (−0.06 to 0.17)	.34	0.21 (0.01 to 0.40)	.04	−0.05 (−0.22 to 0.13)	.60
CD4/CD8 ratio	0.01 (−0.06 to 0.08)	.81	0.09 (−0.03 to 0.20)	.14	−0.10 (−0.18 to −0.02)	.02

^a^All variables were natural log-transformed before analysis.

**Table 5:** Multivariate linear regression models for activity outcomes.

Predictors^a^		Lab-based outcomes		Accelerometer-based outcomes			
		Gait speed		Gait speed		Low activity	
		Coefficient (95% CI)	*P* value	Coefficient (95% CI)	*P* value	Coefficient (95% CI)	*P* value
**Model 1**							
	HIV status	0.04 (−0.15 to 0.07)	.47	−0.20 (−0.39 to −0.02)	.03	−0.15 (0.01 to 0.29)	.04
	Body mass index	−0.35 (−0.67 to −0.36)	.03	−0.59 (−1.13 to −0.06)	.03	0.01 (−0.41 to 0.43)	.96
**Model 2^b^**							
	HIV status^b^	0.12 (−0.17 to 0.26)	.08	−0.10 (−0.37 to 0.16)	.44	0.14 (−0.06 to 0.33)	.17
	Body mass index	−0.32 (−0.61 to −0.02)	.04	−0.65 (−1.22 to −0.07)	.03	0.02 (−0.06 to 0.33)	.94

^a^All variables were natural log-transformed before analysis.

^b^Adjusted for race (ie, white), smoking, and living alone.

## Discussion

### Principal Findings

An emerging challenge in managing PAWH is identifying preclinical sentinel biomarkers to identify changes in the risk for functional decline [35,36]. While consistently associated with adverse health outcomes [6-8], historically, physical function assessments, such as slow walking speed, were designed and validated in older geriatric uninfected populations [7] and may be less sensitive to preclinical features of functional decline in healthier middle-aged individuals. There is, therefore, a need for additional tools for preclinical detection of decline in mobility to prevent the development of disability later in life [12].

In our previously published study of middle-aged HIV+ and HIV− men and women, we observed modest but significant deficits in gait speed, predicted METs, and lab-based stair climb power in HIV+ compared with HIV− men and women [25]. However, when men from that study were analyzed as a subset, despite significantly reduced METs and trend deficits in stair climb power, the differences in gait speed, though lower on average did not reach significance, raising the speculation in this study that lab-based assessment may not fully capture subclinical deficits in mobility emerging in this population. The gait speed obtained during the 6-MWT done under standardized, lab-based conditions did not differentiate performance between HIV+ and HIV− individuals and were not different from published reference values for healthy men of the same age as those in this study [37]. The short duration and controlled conditions under which the 6-MWT is performed may result in faster gait speeds that may not be representative of average and variable daily physical activity and, possibly because of the current reserve capacity of participants, may allow them to compensate for this short-duration assessment [38,39]. In contrast, our measures of physical activity through accelerometry were able to differentiate between groups (*P*=.04), suggesting that this might be a useful preclinical measure of average daily physical activity in this population. As the habitual level of physical activity is associated with physical performance ability (greater physical activity, better physical function), monitoring physical activity may provide a useful marker for identifying preclinical signs of functional decline. The reduced volitional activity observed in this study may reflect diminished physiological energy reserve prior to overt signs of functional deficit.

Prior studies have evaluated PAWH with more advanced inflammation and functional deficits than the current status of participants in this study [5,10]. While HIV+ participants in this study did not differ in a composite score for inflammation (Table 1), they did have elevated levels of the monocyte activation marker sCD163 (data not shown) and did display higher levels of an immune activation composite score and a lower CD4/CD8 ratio, collectively signaling increased risk for multiple comorbid conditions [40], including functional impairment [41]. HIV+ participants reported more pain and increased fatigue (Table 1), consistent with a recent study that showed that pain and exhaustion were predictors of falls in middle-aged HIV+ adults and that functional impairment was associated with elevated immune activation as well as inflammation [4,41].

Conditions, such as depression and pain, which often are identified in PAWH, are known to be associated with the development of frailty [42]. This study shows that men living with HIV were less active than uninfected men when assessed by accelerometers and were also more likely to live alone and experience chronic pain. Social isolation (in this study measured as living alone; Table 1) results in a reduced social network of support and can be a barrier to physical activity that may accelerate the risk for functional decline. Moreover, smoking, common among individuals with HIV [43] may disincentivize activity and exercise because of its negative impact on cardiovascular and pulmonary health [44]. Such modifiable social and behavioral risk factors will need to be further investigated to develop preclinical intervention strategies to prevent disability later in life. Physical activity is an efficacious strategy for the prevention of many chronic conditions, including metabolic, cardiovascular, pulmonary, and musculoskeletal [45], and found to be beneficial for people living with HIV [46-49]. Therefore, a better assessment of activity can improve intervention and monitoring strategies.

A novel aspect of this study was to develop person-specific activity profiles enabled by accelerometry monitoring; this approach may provide individuals with a reference activity pattern that can be used to capture longitudinal changes that signal the development of declines in their reserve that is not yet clinically apparent [50]. Notably, in univariate regression analysis, lab-based gait speed was sensitive to BMI as a predictor, whereas accelerometer-based metrics were sensitive to HIV status, testosterone levels, and CD4/CD8 ratios as predictors (Tables 4 and 5). Despite the small sample size, in a multivariate regression model, BMI and HIV status were independent predictors of accelerometer but not lab-based gait speed (Table 4). However, when adjusted for race, smoking, and living alone, only BMI was an independent predictor of the accelerometer and lab-based gait speed (Table 4), likely because of the small sample size of the study. Collectively, these data suggest that accelerometer- and lab-based physical activity may be linked to physiological conditions that underlie physical activity and may be useful in stratifying individuals into risk groups for future functional limitations.

### Limitations

This study has multiple limitations. The sample size is small and will need confirmation in a larger study. Also, laboratory assessments were performed an average of 16 months prior to activity tracker data collection. However, participants in this study are clinically stable, with an overall accelerometry-based gait speed (0.44 m/s) similar to gait speeds in an independent study sample of over 800 men (0.46 m/s) using Withings trackers in the same age range and sampled randomly across the East Coast (Multimedia Appendix 2). Additional limitations include potential differences in activity because of seasonal variation, physical autonomy, and access to urban greenspaces [51].

### Conclusions

In summary, this study identifies a significantly reduced gait speed and activity pattern in otherwise asymptomatic middle-aged men living with HIV compared with those without HIV, in the absence of detectable differences in physical performance assessed in a laboratory setting. HIV+ participants reported more fatigue and pain, which when coupled with the observed reduced activity may signal a state of preclinical risk for functional decline. Free-living accelerometry may provide a useful biometric tool for monitoring the efficacy of future interventions focused on reducing decline in the physical function in PAWH.

## Appendix

Multimedia Appendix 1 Comparison of the Withings Pulse Ox step count and estimates of gait speed to results from the 6-minute walk test.

Multimedia Appendix 2 Comparison between the mean free-living (volitional) gait speed measured in the MPACT substudy to reference data.

## Abbreviations

6-MWT: 6 minutes walking test
BMI: body mass index
CES-D: Center for Epidemiological Studies Depression
FT: free testosterone
FACIT-F: Functional Assessment of Chronic Illness Therapy-Fatigue
HRQoL: health-related quality of life
MATCH: Muscle Function and Aging in Chronic HIV Infection
MET: metabolic equivalent
PAT-D: Pepper Assessment Tool for Disability
PAWH: People aging with HIV
PROMIS: Patient Reported Outcome Measurements Information System
SHBG: serum hormone-binding globulin
