# Supplementary material for: Middle-Aged Men With HIV Have Diminished Accelerometry-Based Activity Profiles Despite Similar Lab-Measured Gait Speed: Pilot Study
Source: JMIR Mhealth Uhealth. 2019 Feb 1;7(2):e11190. doi: 10.2196/11190 (PMC6376331; doi:10.2196/11190)
Supplement: Multimedia Appendix 1 [file mhealth_v7i2e11190_app1.pdf]

Appendix 1

A test was carried out to compare the Withings Pulse Ox step count and estimates of gait speed to results from the 6-minute walk test (6-MWT). Six members of the study team volunteered to wear the Pulse Ox on their non-dominant wrist and walked for 8 minutes to ensure sufficient accelerometer data was collected to derive an estimate over 6 minutes. Data was collected in the lab along a measured and marked course. The 6MWT assessment is based on counted steps and measurements of the distance covered. The accelerometer estimate is based on number of steps and using the formula (steps*height*0.414)/60).

Step count varied by about ± 100 steps, or ± 15%, with 4 of 6 counts within about 1.5% or less.

Gait speed varied by + 0.22 to – 0/13 mps, or about + 14% to – 10%. 4 of 6 estimates were within 5%.

Table 1. Step Count by Accelerometer (Nokia Pulse Ox) and 6-Minute Walk Test.

Subject	Pulse	6MWT	Difference	% Difference
1	577	682	105	15.40
2	704	609	-95	-15.60
3	674	678	4	0.59
4	642	646	4	0.62
5	671	681	10	1.47
6	699	700	1	0.14

Table 2. Estimated Gait Speed by Accelerometer (Nokia Pulse Ox) and 6-Minute Walk Test.

Subject	Pulse	6MWT	Difference	% Difference
1	1.15	1.20	0.05	4.33
2	1.38	1.60	0.22	13.99
3	1.34	1.40	0.06	4.21
4	1.20	1.20	0.00	-0.28
5	1.31	1.30	-0.01	-0.90
6	1.43	1.30	-0.13	-10.08
