# Supplementary material for: Middle-Aged Men With HIV Have Diminished Accelerometry-Based Activity Profiles Despite Similar Lab-Measured Gait Speed: Pilot Study
Source: JMIR Mhealth Uhealth. 2019 Feb 1;7(2):e11190. doi: 10.2196/11190 (PMC6376331; doi:10.2196/11190)
Supplement: Multimedia Appendix 2 [file mhealth_v7i2e11190_app2.pdf]

Appendix 2

Withings (co-author ER) extracted 1 year of accelerometer data (monthly average step counts) for adults living in the US Eastern time zone. The anonymous data included 813 men, ages 50-65 years (mean = 56.83) and height in meters from 1.57 to 2.97 (mean = 1.79) . The formula (steps*height*0.414)/60) was used to create an estimate of gait speed in meters per second.

A two-sided t-test was used to compare the mean free-living (volitional) gait speed measured in the MPACT sub-study to the reference data. *P*-values are listed in the table below.

Table 1. Mean free-living (volitional) gait speed assessed by accelerometer.

Data	Gait Speed (mps)	SD	Min.	Max.	*P*-value[a]
Reference Data (n=813)	0.464	0.180	0.085	1.468	NA
MPACT (n=46)	0.441	0.133	0.220	0.838	.40
MPACT HIV+ (n=23)	0.406	0.115	0.220	0.669	.13
MPACT HIV- (n=23)	0.476	0.142	0.260	0.838	.75

a. Two-sided t-test comparing MPACT mean gait speed to the reference data gait speed.
